# A New Method for Identification of Ginseng Radix et Rhizoma Adulterated with Panacis Quinquefolii Radix

**DOI:** 10.3390/foods14203566

**Published:** 2025-10-20

**Authors:** Yihang He, Xinyue Zhang, Zhe Wu, Wen Li, Lihui Zhang, Jiating Zhang, Fangliang He, Jia Chen, Xianlong Cheng, Feng Wei

**Affiliations:** 1 Institute for Control of Traditional Chinese Medicine and Ethnic Medicine, National Institutes for Food and Drug Control, Beijing 102629, China; heyihang0302@outlook.com (Y.H.); zhangxinyue@nifdc.org.cn (X.Z.); wuzhe@nifdc.org.cn (Z.W.); lw20011006@163.com (W.L.); 13199634075@163.com (L.Z.); 18341441178@163.com (J.Z.); hefangliang0602@126.com (F.H.); chenjia@nifdc.org.cn (J.C.); 2 China Medical University-Queen’s University of Belfast Joint College, China Medical University, Shenyang 110100, China

**Keywords:** ginseng radix et rhizoma, panacis quinquefolii radix, matrix identity cards, quality control, UPLC-QTOF-MS, contrast credibility

## Abstract

In the regulatory market, it is not uncommon for ginseng radix et rhizoma (GR) to be adulterated with panacis quinquefolii radix (PR). Amid the digital transformation, this study puts forward a new method for the identification of GR adulterated with PR. Ultra-high-performance liquid chromatography–quadrupole time-of-flight mass spectrometry (UPLC-QTOF-MS) was used to detect multiple batches of GR and PR to obtain mass spectrometry data. The common ions were isolated from multiple batches of GR and PR, serving as GR and PR’s “ion matrices”. Furthermore, GR and PR’s “ion matrices” were used to eliminate intersecting ion data to extract the top-100 ions as GR and PR “matrix identity cards” (MICs). Then, GR and PR’s MICs were employed as a reference for identification, yielding contrast credibility (CC) as feedback. The results indicated that leveraging the MICs of GR and PR enables efficient and precise digital identification of the two herbs: pure GR showed CC ≥ 95% when matched with GR MIC (≤2% with PR MIC), pure PR showed CC ≥ 93% with PR MIC (≤3% with GR MIC), and non-parametric analysis confirmed significant differences between groups (*p* < 0.01). Even in 5% PR-adulterated samples, CC ranged from 24% to 28% (avg. 25.8%) when matched with PR MIC, leading to a 26% adulteration detection threshold. Moreover, two adulterated batches were identified among ten GR blind samples, which was consistent with verification via PR-specific pseudo-ginsenoside F_11_. This research is practically valuable for distinguishing between GR and PR, combating adulteration, and reinforcing GR quality management. It also informs the digital identification of GR via UPLC-QTOF-MS and “MICs”, contributing to the digital quality control of traditional Chinese medicines (TCMs).

## 1. Introduction

Ginseng radix et rhizoma (GR) and panacis quinquefolii radix (PR), as representative medicinal plants of the *genus Panax* in the Araliaceae family, hold a pivotal position in global traditional medicine systems [1]. Both are used medicinally with their rhizomes and are regarded as precious herbs in traditional Chinese medicine, with their medicinal history dating back thousands of years [2]. Modern pharmacological studies have further confirmed that the bioactive components, such as ginsenosides, polysaccharides, and amino acids, in PG and PL possess various biological activities, including regulating central nervous system functions, improving cardiovascular health, and enhancing immune activity [3,4]. Consequently, their applications in the fields of pharmaceuticals, health products, and functional foods have become increasingly widespread, with the market demand continuing to climb [5].

However, GR and PR not only have a close phylogenetic relationship but also share highly similar morphological characteristics—their fresh rhizomes are fleshy, mostly cylindrical, or spindle-shaped, with dense fine wrinkles on the surface; after drying and processing, their colors both tend to be yellowish-white to light brown, making accurate differentiation merely through visual observation extremely difficult. More importantly, their chemical compositions have significant overlaps, with ginsenosides as the main active components. This greatly reduces the accuracy of traditional physical and chemical identification methods. At the same time, there are frequent occurrences of adulteration and counterfeiting in the market: some merchants pass off PR as GR to reduce costs, as the market price of GR is generally higher than that of PR, or mix PR into GR for gain [6,7].

In recent years, liquid chromatography–mass spectrometry (LC-MS) has become a core tool in the component analysis and identification of traditional Chinese medicines (TCMs) due to its high resolution, high sensitivity, and high specificity [8,9,10,11]. Among these analytical techniques, ultra-high performance liquid chromatography–quadrupole time-of-flight mass spectrometry (UPLC-QTOF-MS) can achieve the separation and identification of hundreds of components in complex systems within a short time. It has superior separation efficiency and mass accuracy, enabling the capture of low-abundance or trace characteristic ions in complex matrices [12,13].

Furthermore, compared with traditional identification methods, digital identification of TCMs has the advantages of strong objectivity, high repeatability, and facilitation of automated analysis, which is more in line with the needs of modern quality control [14,15]. Its core lies in extracting the chemical information of TCMs and constructing a digital identification system, thereby realizing the transformation from “morphological identification” to “data identification” [16]. Previously, although some studies have combined LC-MS with chemometric methods (Principal Component Analysis (PCA), Orthogonal Partial Least Squares-Discriminant Analysis (OPLS-DA), etc.) or machine learning methods for the identification of GR and PR [17,18], these methods mostly have problems, such as lack of quantitative indicators, weak detection ability for low-level adulteration, and poor anti-interference performance, resulting in limited applicability and reproducibility.

With the further advancement of the digitalization wave in TCMs, the construction of a more standardized and operable digital identification method has become a key demand. To this end, this study further develops the methodology on the basis of previous digital identification methods and proposes a new method for identifying GR adulterated with PR: first, with UPLC-QTOF-MS serving as the data acquisition platform, common ions from multiple batches of GR and PR are extracted through digital conversion to construct an “ion matrix”; then, species-specific ions are obtained through differential screening, and the top 100 ions ranked by ionic intensity are innovatively selected to construct “matrix identity cards (MIC)”. Meanwhile, this study introduces “contrast credibility (CC)” as a quantitative evaluation index for the first time, and it defines a clear CC detection threshold through the verification of multiple batches of samples adulterated with 5% PR. Through the standardized construction of MIC and the quantitative feedback of CC, this method enables the accurate identification of GR and PR, as well as the detection of adulteration, providing a more practically valuable new framework for the digital quality control of TCMs.

## 2. Experiment Consumables and Research Methods

### 2.1. Experiment Consumables

In total, 15 GRs and 15 PRs were collected from Liao Ning, Hei Long Jiang, Ji Lin, Hebei, and Shandong Provinces, China. More than 30 batches of medicinal materials were identified by Wei Feng, a researcher, and all samples met the standard requirements in Ch.P [19]. In total, 11 batches of positive adulterated samples (PASs) were prepared with different proportions of GR and PR by ourselves. In total, 10 GR blind samples were purchased in a herbal medicine market. Table S1 shows 51 batches of herbal material information. Wahaha Co., Ltd. (Hangzhou, China), provided purified water (Lot: 220337TH). MS-grade acetonitrile (Lot: R142677), MS-grade methanol (Lot: R142435), and formic acid (Lot: 8387760) were all bought from Sigma Aldrich Co., Ltd. (Shanghai, China). Disposable sterile syringes (2 mL, lot: DC02100) were obtained from Shanghai Anpel Experimental Technology Co., Ltd. (Shanghai, China). Waters sampling vials (Lot:GB19298) were purchased from the Waters Corporation (Milford, MA, USA). MS filter membranes (Lot: F243359) were sourced from Beijing Dikma Technology Co., Ltd. (Beijing, China) Pseudo-ginsenoside F11 (Lot: 110841-202410, 98.1%) was purchased from the National Institutes for Food and Drug Control (Beijing, China).

### 2.2. Operational Processing

The GR and PR were first sliced, pulverized using a high-speed grinder, and passed through a No. 3 sieve. Equal aliquots of powder from each batch were mixed to prepare mixed samples of GR and PR. Positive adulterated samples with 0%, 5%, 10%, 20%, 50%, and 100% PR were then prepared by blending the PR mixed sample into the GR mixed sample. Subsequently, 0.50 g of powder from each batch of GR, PR, and positive adulterated samples was accurately weighed using an XS-105DU balance (Lot :B742830549). Each weighed powder was transferred to a 25 mL stoppered conical flask, followed by the precise addition of 25.00 mL of 50% methanol. After shaking for 30 s, the mixture was sonicated for 30 min (500 W; 40 kHz). The mixture was then removed, cooled to room temperature (25 °C), and filtered through an MS filter membrane.

### 2.3. Mass Spectrometry Conditions

The analyses were performed using a Waters UPLC and Synapt G2-S Q-TOF MS system (Milford, MA, USA). The liquid chromatography and mass spectrometry conditions are specified as follows: for liquid chromatography, a Waters Acquity^TM^ UPLC BEH C_18_ column (2.1 mm × 50 mm, 1.7 μm, Serial No. 04553318718652) was employed. The mobile phase consisted of acetonitrile (A) and 0.1% formic acid aqueous solution (B), with a gradient elution program as follows: 5–17% A at 0–5 min; 17% A at 5–5.5 min; 17–19% A at 5.5–10 min; 19–23% A at 10–12 min; 23–26.5% A at 12–13.5 min; 26.5–30% A at 13.5–20 min; 30–50% A at 20–25 min; 50–65% A at 25–33 min; 65–95% A at 33–33.1 min; 95% A at 33.1–37 min; 95–5% A at 37–37.1 min; and 5% A at 37.1–41 min. The column temperature was maintained at 40 °C, sample injection was 5 μL, and liquid phase flow velocity was 0.8 mL/min. For MS conditions, ESI^–^ and MS^E^ were adopted. Cone voltage was 40 V, and the collision voltage ranged from 10 to 50 V. Capillary voltage was 2.5 kV, and source offset voltage was 80 V. Acquisition rate was 0.2 s, and the desolvation temperature was maintained at 300 °C. The scan range was 100–1200 Da. The source temperature and flow rate of desolvation gas were 600 L/h and 120 °C. Calibration interval was 30 s.

### 2.4. Data Transformation and “Matrix Identity Cards”

For the MS results for GR and PR, data transformation was performed using the Waters Progenesis QI analytical software (version number 2.4.69), which is specifically designed for MS analysis. The data conversion was as follows [15,20]:

A high resolution was utilized as the analytical condition, operating in negative ion mode with an ionization source for ionization. Moreover, the peak detection threshold was configured for automatic mode. The retention time (*t_R_*) window was 1.00–33.00 min. Using these parameters, quantitative data were obtained for analysis, encompassing *t_R_*, *m*/*z*, and I. These quantitative data were then saved as a data matrix (DM) in “CSV files”. After the data were finally collated, they were placed in the following matrix form [14,15,21]:

$$\left[\begin{array}{ccc} t_{R} & m/z & I\\ \cdots & \cdots & \cdots \\ t_{n} & m_{n} & i_{n} \end{array}\right]$$

**Figure 1:**
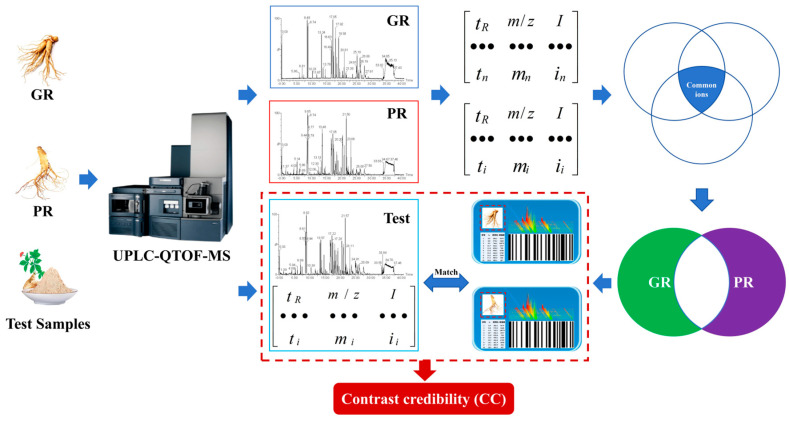
The GR and PR “matrix identity card” acquisition and matching process.

Based on the data matrix of blanks, GR, and PR, the GR and PR “matrix identity cards” and matches can be obtained through the process in Figure 1 [22,23].

(1) Blank ion subtraction: Interfering ions derived from 50% methanol–water were present in all samples. If the ions in the samples and the blank had similar *t_R_* and *m*/*z* values, the ions would be removed.

(2) Data matrix of common ions: The common ions from different batches of the same Chinese medicine—GR or PR—were extracted by taking the intersection to construct new data matrices of GR and PR. Similarly, the common ions have similar *t_R_* and *m*/*z* values.

(3) “Matrix identity cards”: Furthermore, the data matrices of specific ions from GR and PR were extracted through difference set processing, where if the ions in both the GR and PR’s common ions have similar *t_R_* and *m*/*z* values, the ions would be identified as non-proprietary ions and thus excluded. Finally, we sorted by ionic strength from highest to lowest, and uniformly took the data matrix containing the top 100 ions as GR and PR “matrix identity cards”.

(4) Digital identification: the GR and PR’s “matrix identity cards” were employed as a reference for identification, yielding a contrast credibility (CC) as feedback. If the ions detected in the test sample are similar to those in the “MICs”, the match is successful, and the similar ions have similar *t_R_* and *m*/*z* values.

$$\mathrm{CC}=\frac{number\;of\;matched\;ions}{number\;of\;ions\;in\;matrix\;identity\;card}\times 100\%$$

In the steps described above, the *t_R_* and *m*/*z* values of similar ions refer to Δ*t_R_* ≤ 0.20 min and Δ*m*/*z* ≤ 0.01 Da.

## 3. Experiment Results and Analysis Discussions

### 3.1. Mass Spectrometry Experiment

**Figure 2:**
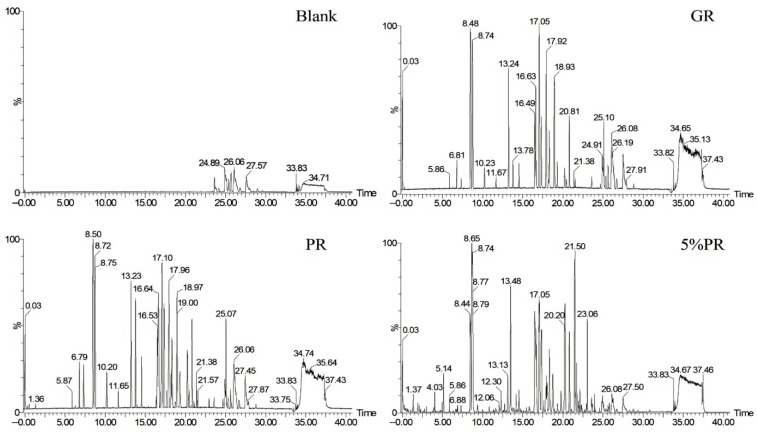
Mass spectrograms of blank, ginseng radix et rhizoma (GR), panacis quinquefolii radix (PR), and positive adulterated sample (5%PR).

Figure 2 shows an MS spectrogram of the blank, GR, PR, and PASs under the same conditions. As shown in Figure 2, the blank solvents do not significantly interfere with the MS analysis of the samples. Interestingly, since both GR and PR belong to the Araliaceae family, their main active components contain various types of ginsenosides. Moreover, after processing them into slices, the key discriminatory characteristics disappear, resulting in higher similarity in appearance. It can be stated that the mass spectral differences between GR and PR are not obvious, making it difficult to distinguish them by visual inspection of a mass spectrogram alone. Furthermore, if PR is adulterated in GR, it becomes even more challenging to judge authenticity solely based on mass spectral images. Fortunately, the continuous development of digital technologies for TCMs has provided significant assistance in this regard.

On the other hand, prior to MS acquisition of GR and PR, sample processing was explored under liquid chromatography (LC) detection conditions and MS detection conditions. The selection of 50% methanol as the extraction solvent was based on the fact that more MS data were obtained compared to the attempts using methanol, ethanol, and 70% methanol. Subsequently, the LC detection conditions were adjusted, and we found that using 0.1% formic acid resulted in relatively delayed retention times and a strong response compared to 0.3% formic acid, which is beneficial due to the characteristics of less peak overlapping and higher intensity. Meanwhile, we debugged the MS detection conditions and compared the results under positive and negative ion modes. It was observed that ion numbers were generally higher in negative ion mode than in positive ion mode. In summary, the resulting detection method is more conducive to the establishment of MICs for GR and PR.

### 3.2. Data Transformation

Methanol was utilized as the “blank control” to subtract background ions. Following data processing and transformation via the Progenesis QI software, the count of [*t_R_*-*m*/*z*-I] units therein fell within a range of 3679 to 4425 [17,18]. Regarding the GR and PR standard samples employed for extracting “MICs”, Table 1 shows the specific quantity information of the [*t_R_*-*m*/*z*-I] units.

As exemplified in Table 1, 10 GR and 10 PR samples were randomly selected for the extraction of “MICs”. Samples of GR and PR from distinct batches displayed variations in the quantity of [*t_R_*-*m*/*z*-I]. To illustrate, the GR sample with the batch number GR01 contained 3679 [*t_R_*-*m*/*z*-I], whereas the GR sample labeled GR05 had 3954 such units. In the case of PR samples, batch PR01 contained 3740 [*t_R_*-*m*/*z*-I] units, while batch PR04 had 4218 units. These observations reveal that significant differences in the quantity of [*t_R_*-*m*/*z*-I] exist between different GR and PR samples, indicating inconsistencies in the mass spectral information across batches, which essentially stem from differences in chemical compositions. Such compositional variations may arise from the combined effects of multiple factors, including different regions, sample storage duration, and individual differences, aligning well with practical scenarios. Additionally, given the unavoidable differences in various batches and the origins of the same herbs, it is imperative to comprehensively consider the various batches and origins of GR and PR to gain their “matrix identity cards”.

### 3.3. “Matrix Identity Cards” of GR and PR

Through digital processing, 10 GR and 10 PR samples, as listed in Table 1, were chosen to calculate “matrix identity cards”. Based on the MIC acquisition process, 100 [*t_R_*-*m*/*z*-I] entities served as “matrix identity cards” for GR and PR. Table S2 shows the “MICs” of GR, and Table S3 shows the “MICs” of PR.

In capturing GR and PR’s “MICs”, the initial *t_R_* deviation threshold was set to Δ*t_R_* ≤ 0.05 min. The initial *m*/*z* deviation threshold was set to Δ*m*/*z* ≤ 0.01 Da. Under such rigorous criteria, however, only a few ions survived the screening. A subsequent adjustment to Δ*t_R_* ≤ 0.10 min with Δ*m*/*z* remaining at 0.01 Da resulted in an insufficient number of PR-specific ions—even failing to reach the 100-ion benchmark—though this did enable more precise selection based on ion intensity. When the *t_R_* threshold was modified to Δ*t_R_* ≤ 0.20 min and the *m*/*z* threshold was Δ*m*/*z* ≤ 0.05 Da, the count of PR-proprietary ions increased, and the detection accuracy was compromised: significant mismatches emerged during identification, giving rise to false-positive outcomes. Following thorough evaluation and iterative testing of various parameter combinations, the optimal threshold for *t_R_* was determined to be Δ*t_R_* ≤ 0.20 min. The *m*/*z* threshold was determined to be Δ*m*/*z* ≤ 0.01 Da.

### 3.4. Identification of GR and PR

With the “MICs” of GR and PR serving as benchmarks, the test sample’s data matrix was matched in sequence to yield CC values. A total of 10 test sample batches were prepared, encompassing five GR and five PR samples. Table 2 shows the matching results for GR.

As shown in Table 2, compared with the “MICs” of GR, the CC values of all GR samples reached an excellent level of 95%. Among them, the CC value of GR04 reached a maximum of 101%, which is attributed to data deviation. In contrast, when compared with the “MICs” of PR, the CC values of all PR samples did not exceed 0%. In other words, there is a significant discrepancy between the CC values of GR and its own “MICs” versus those between GR and the PR “MICs”. Table 3 shows the PR matching results. Compared with the “MICs” of PR, the CC values of all PR samples reached an excellent level of 93%, with the CC values of PR06 and PR11 reaching a maximum of 100%. In contrast, compared with the GR “MICs”, the CC values of all PR samples did not exceed 3%. Furthermore, the CC values of GR and PR that matched their own “MICs” were divided into the “A” group, and the CC values of GR and PR matched with non-self “MICs” were divided into the “B” group for statistical analysis. Table 4 shows the results of the non-parametric statistical analysis. *p* = 0 < 0.01 demonstrates that the CC in group A and group B had significant statistical differences. Therefore, these two easily confused medicinal herbs—GR and PR—can be effectively distinguished using “MICs”. The results not only further verify the reliability of “MICs” in distinguishing the two medicinal herbs but also highlight the stable consistency demonstrated in the detection of samples from different batches. Even when there is a certain fluctuation in CC values, the GR04 batch reaches 101%, and the overall data remains firmly within the high-specificity range, indicating that “MICs” will not lead to misjudgement due to individual differences in samples.

Meanwhile, the CC values in the comparison of the “MICs” between PR and GR were always below 3%, which forms a sharp contrast with the high values when PR and GR were matched with their own “MICs”. From the reverse perspective, this confirms that the uniqueness boundaries of the “MICs” of the two medicinal herbs are clear. Moreover, the CC value gap of more than 30 times intuitively demonstrates that there is an order-of-magnitude difference in the ability of “MICs” to identify con-specific and hetero-specific medicinal herbs. In addition, combined with the detection performance in Table 2 and Table 3, it is not difficult to see that both GR and PR “MICs” show extremely high consistency in self-matching and maintain extremely low cross-reactivity in cross-species comparison. This two-way specificity characteristic lays a solid foundation for the digital identification of GR adulterated with PR.

### 3.5. Analysis of Positive Adulterated Samples

**Figure 3:**
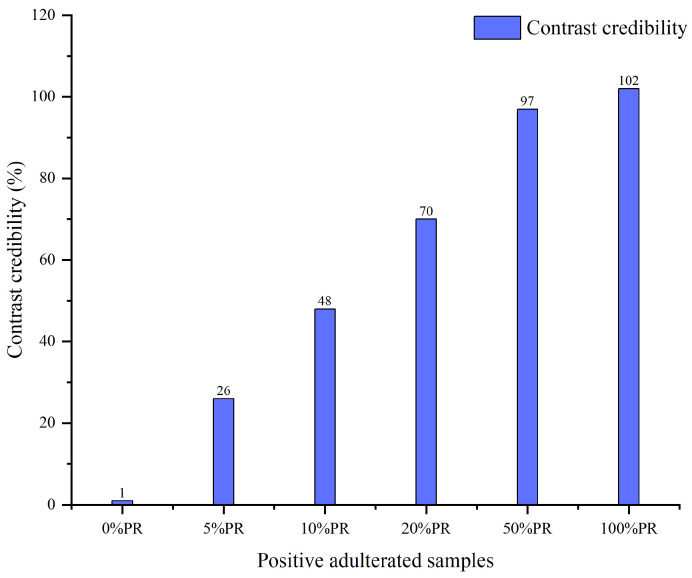
The matching results of positive adulterated samples compared with the “matrix identity cards” of panacis quinquefolii radix.

The “MICs” of GR and PR were used to match the positive adulterated samples. Figure 3 presents the matching results between the positive adulterated samples (PASs) and the PR “MICs”.

**Figure 4:**
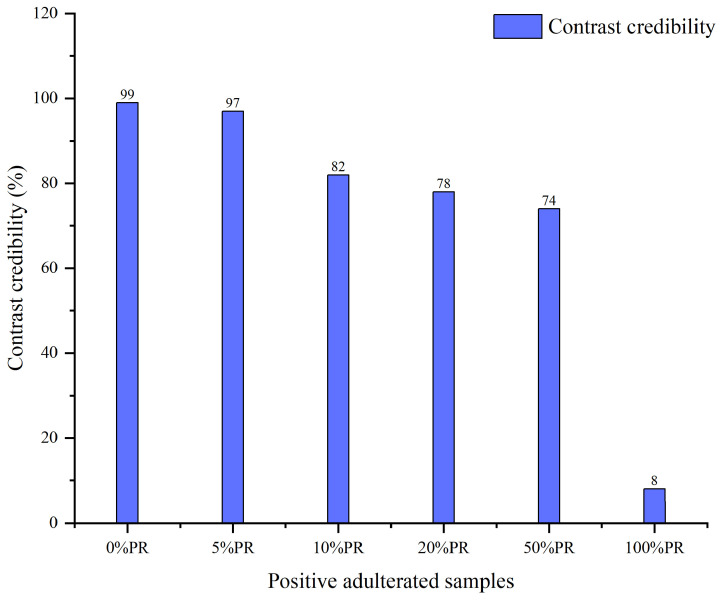
Matching results for positive adulterated samples compared with “matrix identity cards” of ginseng radix et rhizoma.

As illustrated in Figure 3, as the proportion of PR in the PAS rises, the CC corresponding to the GR “MICs” exhibits a generally upward pattern. When the PR proportion ranges from 0% to 5%, the CC values relative to PR “MICs” are 1% and 26%, which exist as a 25-times difference and have obvious recognition. As the PR proportion further increases from 10% to 50%, the CC values increase successively to 48%, 70%, and 97%. Furthermore, the CC of the 100% PR sample compared with the PR “MICs” is 102%. The above results indicate that the GR in PASs can be effectively identified by relying on PR “MICs”. Conversely, Figure 4 presents the matching outcomes of the PASs with respect to GR “MICs”. As the proportion of PR in the PASs rises, the CC corresponding to the GR “MICs” exhibits a generally decreasing pattern. When the PL proportion ranges from 0% to 50%, the CC values relative to the GR “MICs” all exceed 73%. Notably, the CC of the 100% PR sample compared with the GR “MICs” is merely 8%. The above results demonstrate that PR in PASs can be successfully identified on the basis of PR “MICs”, even when the PAS contains only 5% PR. Moreover, this does not affect the identification of GR in PASs.

In summary, the digital analysis of GR, PR, and PASs confirms that the “MICs” of both GR and PR possess a certain degree of specificity. The specificity of “MIC” enables the digital analysis of PG and PL at the TCM level and facilitates the detection of adulterated samples. The CC serves as a crucial feedback indicator, allowing for rapid and reliable digital identification based on its values. Combining Figure 3 and Figure 4, it is evident that for a sample with 0% PR (GR), its CC compared with GR “MICs” is as high as 99%, whereas compared with PR “MICs”, its CC is only 1%. Therefore, for cases of PR adulteration in GR samples, we can set the detection threshold for CC according to the matching results based on PR “MICs”. Furthermore, considering that impurities are allowed in TCMs, 5% PR is used as the adulteration limit to set the detection threshold of CC.

Considering the deviation threshold and the natural fluctuation of the detection data, it is necessary to prepare multiple positive adulterated samples with 5% PR in parallel to set the CC threshold. Table 5 shows the CC results for 5% PR-adulterated samples (PASs). The CCs range from 24% to 28%, and their average value is 25.8%. Finally, the average value (26%) is used as the CC threshold based on PR “MICs”. In the aforementioned analysis, we prepared multiple 5% PASs to establish detection limits. As determined by averaging multiple measurements, the average value, as a representative value, can be used to summarize the central tendency of a data set and effectively reduce random error. As shown in Table 5, these CCs do not exhibit interference from extreme values, so the average value is the most representative. It is worth noting that the CC value of the adulteration detection threshold is not constant. It depends on the positive adulteration control samples prepared during test analysis, just as the accompanying standard curve does in quantitative analysis. Therefore, in subsequent practical analyses, it is necessary to prepare corresponding positive adulterated samples to assist in the determination of the threshold.

### 3.6. Analysis of GR Blind Samples

**Figure 5:**
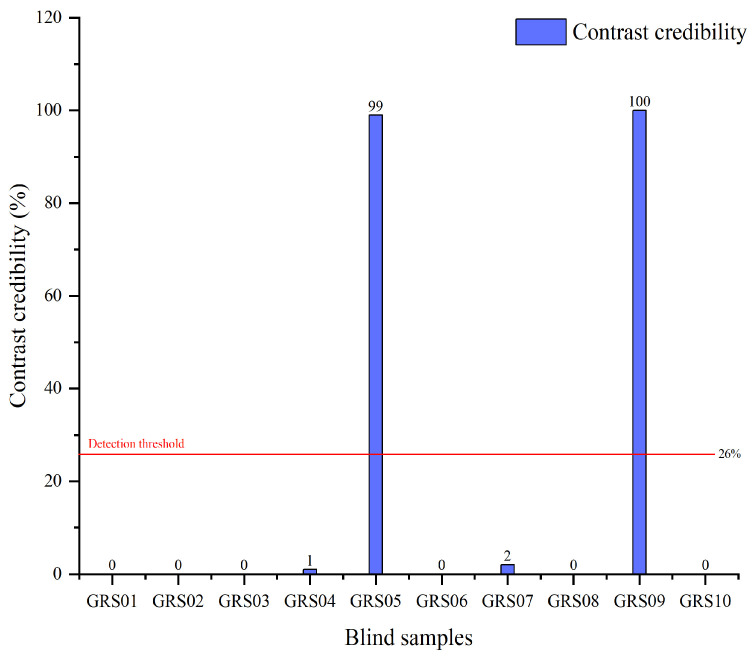
The matching results for blind samples compared with “matrix identity cards” of ginseng radix et rhizoma.

Furthermore, a matching analysis between 10 batches of GR blind samples (GRSs) and the GR and PR “MICs” was conducted in this paper. Figure 5 shows the matching results between the GRSs and the “MICs” of PR. The findings revealed that, except for GRS05 and GRS09, the CC values of the remaining samples (GRS01, GRS02, GRS03, GRS04, GRS06, GRS07, GRS08, and GRS10) were all less than 3%, which is far below the CC detection threshold of PR (26%). However, the CC values of GRS05 and GRS09 were 99% and 100%, which are far higher than the CC detection threshold of PR (26%). In other words, based on “MIC” matching, we proved that the GRS05 and GRS09 samples were adulterated with PR. The remaining samples can be identified as authentic ginseng radix et rhizoma. On the other hand, Table 6 shows the CC results between the GRSs and the “MICs” of GR. The CC values of all blind samples were greater than 67%, which was far higher than the CC value of the PR mix sample compared with the GR “MICs” (8%). This did not affect the identification of GR.

### 3.7. Matching Result Verification

**Figure 6:**
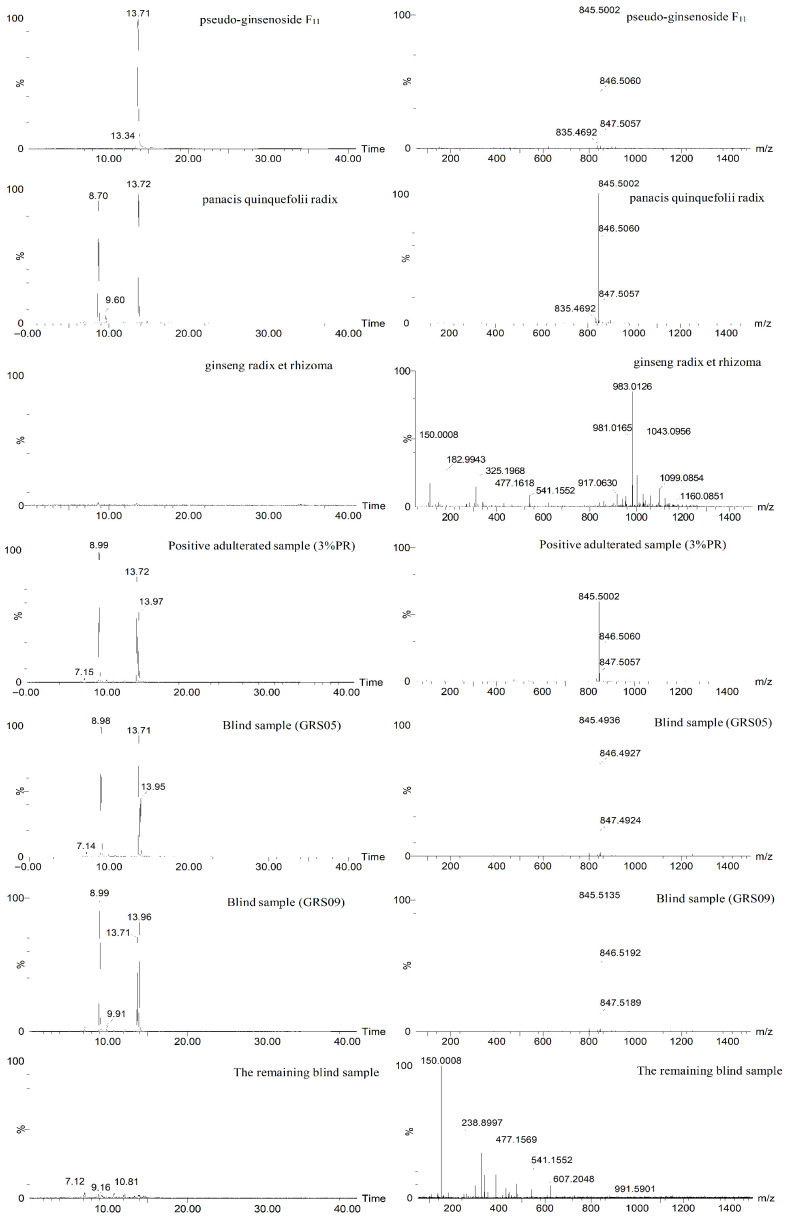
The extraction situation for the proprietary chemical component of panacis quinquefolii radix—pseudo-ginsenoside F_11_.

Although the analysis of positive adulterated samples and blind samples proves that MIC-based identification analysis is reasonable and reliable, the new method still needs to be verified. As we all know, pseudo-ginsenoside F_11_ (C_42_H_72_O_14_, CAS: 69884-00-0) is the proprietary chemical component of PR [24]. Therefore, the new method can be validated based on the extraction of pseudo-ginsenoside F_11_. In ESI^–^ mode, pseudo-ginsenoside F_11_ is created either by losing one hydrogen ion [M–H]^–^ or by combining with an acid ion [M + HCOO]^–^. Figure 6 shows the [M + HCOO]^–^ extraction situation of pseudo-ginsenoside F_11_ (13.71 min_845.50 *m*/*z*).

As illustrated in Figure 6, the proprietary ion of 13.71 min_845.49 *m*/*z* can be detected in the MS information of PR but cannot be detected in the MS information of the GR samples. For positive adulterated samples, the proprietary ion of 13.71 min_845.49 *m*/*z* can be detected even in the 5% PR-adulterated sample. As far as the GR blind samples are concerned, the proprietary ions of PR can be extracted from the MS information of GRS05 and GRS09, but they cannot be checked in the other GR blind samples. The above results indicated that PR was indeed adulterated in the GRS05 and GRS09 blind samples, which is consistent with the identification results based on the new method—“MICs”. This strongly verifies that “MICs” have certain specificity, and the new identification method based on “MICs” can realize the identification of GR-adulterated PR.

### 3.8. The Significance and Restrictions of “Matrix Identity Cards”

Digital identification technologies play a pivotal role in addressing long-standing challenges in the field of TCMs. Traditional identification methods, reliant primarily on morphological characteristics and subjective experiential judgment, are inherently limited by variability in expertise and environmental factors, leading to high rates of misclassification. In contrast, digital identification, encompassing techniques such as hyperspectral imaging, mass spectrometry-based metabolomics, and machine learning-driven pattern recognition, enables the objective, high-throughput, and standardized authentication of TCM materials [25,26,27,28,29]. Therefore, against the background of the digital age, this article introduces the concept of “matrix identity cards”. “Matrix identity cards” can be used to fully consider the common chemical components of GR (or PR) from different batches and different places and to fully consider the different chemical components of GR and PR. More notably, compared with a single proprietary chemical component—pseudo-ginsenoside F_1_—the quantitative matrix of chemical components—“matrix identity cards”—contains many proprietary chemical components that have stronger specificity.

Compared with the previous identification processes—“matrix characteristics” and adulteration analysis of GR and PR [15,19,28]—“MICs”, constructed by screening specific ions from multi-batch samples and introducing CC as an evaluation index, demonstrate strong objectivity and repeatability. Unlike traditional physical and chemical methods that rely on experience, “MICs” convert chemical information into quantifiable digital matrices, reducing human error and standardizing the identification process. Their high sensitivity, enabled by UPLC-QTOF-MS’s ability to capture ion information, allows for the detection of subtle chemical differences between GR and PR that are undetectable in visual inspection or conventional analysis. In practical analysis, “MICs” enhance the efficiency of distinguishing between GR and PR, providing a new method to combat adulteration and counterfeiting in the market, thereby strengthening the quality management of these medicinal materials. This also contributes to the digital transformation of TCM by offering a framework for a new digital identification method based on “MICs”, supporting the development of digital quality control for GR.

Although GR and PR samples were primarily collected from multiple regions in China, this study is constrained by sample size and scale. In addition, this study did not conduct cross-platform application practices. Therefore, future work could expand the sample size and scale to include more geographical origins (e.g., the USA or North Korea) and different cultivation conditions, so as to improve the generalizability of “MICs” for GR and PR and conduct cross-platform application research based on the relative retention time method and mass-to-charge ratio calibration. Moreover, this research should integrate artificial intelligence algorithms to optimize the matching process and develop intelligent systems and equipment. In addition, extending “MICs” to other herbal medicines will further promote the digitalization and intellectualization of TCM quality control.

## 4. Conclusions

In the paper, a new method— “matrix identity cards”—based on UPLC-QTOF-MS was successfully constructed to identify PR-adulterated GR. Furthermore, two blind samples were identified as PR-adulterated samples. The “matrix identity cards” of GR and PR have certain proprietary characteristics that facilitate the digital analysis of GR adulterated with PR. This contributes to the digital quality control of GR. This research transcends the constraints of traditional identification methods, offering a replicable framework for authenticating other easily confused TCMs. For samples flagged as “suspected adulterated” in primary screening, this method can be combined with species-specific component validation (e.g., pseudo-ginsenoside F_11_ for PR) to form a “primary screening–verification” two-step workflow. This not only maintains the efficiency of initial screening but also ensures the accuracy of final results, making it suitable for high-stakes scenarios such as dispute resolution in regulatory cases or raw material acceptance in pharmaceutical production. By enabling the future development of a broad MIC database, this method lays the foundation for a “Digital Identification System for TCMs” that will help ensure drug safety for consumers and regulate the market. More importantly, it plays a pivotal role in transitioning TCM quality control from traditional to digital paradigms, thereby facilitating industry-wide standardization and supporting global acceptance through the provision of objective quality standards. In summary, the UPLC-QTOF-MS-MIC method constructed in this study not only provides a reliable tool for combating GR adulteration and protecting consumer medication safety but also accelerates the transformation of TCM quality control from a “traditional experiential paradigm” to a “digital data-driven paradigm”. By providing objective, quantitative, and standardized identification indicators, it lays a solid foundation for the standardization and internationalization of the TCM industry.

## Figures and Tables

**Table 1:** The count of [*t_R_*_-_*m*/*z*-I] entities in samples utilized for the extraction of “MICs”.

Samples	Batches	Number	Samples	Batches	Number
Ginseng radix et rhizoma (GR)	GR01	3679	Panacis quinquefolii radix (PR)	PR01	3740
	GR03	3707		PR02	3918
	GR05	3954		PR04	4218
	GR07	4036		PR05	4309
	GR08	4166		PR07	4425
	GR09	3760		PR09	3832
	GR10	4216		PR10	4173
	GR12	3853		PR11	4218
	GR14	3752		PR12	4279
	GR15	4025		PR14	4385

**Table 2:** The contrast credibility of the GR-matching situation.

Herb Name	Batches	Matching Successful Ions	Ion Number in MICs	CC
Ginseng radix et rhizoma (GR)	GR02	99	100-GR	99%
	GR04	101	100-GR	101%
	GR06	100	100-GR	100%
	GR11	98	100-GR	98%
	GR13	95	100-GR	95%
	GR02	0	100-PR	0%
	GR04	0	100-PR	0%
	GR06	0	100-PR	0%
	GR11	2	100-PR	2%
	GR13	0	100-PR	0%

**Table 3:** The contrast credibility of the PR-matching situation.

Herb Name	Batches	Matching Successful Ions	Ion Number in MICs	CC
Panacis quinquefolii radix (PR)	PR03	93	100-PR	93%
	PR06	100	100-PR	100%
	PR08	96	100-PR	96%
	PR11	100	100-PR	100%
	PR13	98	100-PR	98%
	PR03	1	100-GR	1%
	PR06	3	100-GR	3%
	PR08	2	100-GR	2%
	PR11	1	100-GR	1%
	PR13	0	100-GR	0%

**Table 4:** The results of the non-parametric test.

Class	Median		Mann–Whitney-*U*	Mann–Whitney-*Z*	*p*
	Group A (*n* = 10)	Group B (*n* = 10)			
CC	98.5%	0.5%	0	−3.819	0.000 **

** *p* < 0.01.

**Table 5:** The contrast credibility of 5% PR-adulterated samples.

Herb Name	Batches	Matching Successful Ions	Ion Number in MICs	CC	Average
Adulterated samples (5% PR)	PR03	28	100-PR	28%	25.8%
	PR06	26	100-PR	26%	
	PR08	26	100-PR	26%	
	PR11	24	100-PR	24%	
	PR13	26	100-PR	25%	

**Table 6:** The contrast credibility of blind samples compared with GR “MICs”.

Herb Name	Batches	Matching Successful Ions	Ion Number in GR MICs	CC
Blind samples	GRS01	92	100-GR	92%
	GRS02	94	100-GR	94%
	GRS03	98	100-GR	98%
	GRS04	102	100-GR	102%
	GRS05	67	100-GR	67%
	GRS06	87	100-GR	87%
	GRS07	89	100-GR	89%
	GRS08	93	100-GR	93%
	GRS09	71	100-GR	71%
	GRS10	92	100-GR	92%

## Data Availability

The original contributions presented in this study are included in the article/ Supplementary Materials. Further inquiries can be directed to the corresponding authors.

## Supplementary Materials

The following supporting information can be downloaded at https://www.mdpi.com/article/10.3390/foods14203566/s1: Table S1: Detailed information of herbal materials. Table S2: “Matrix identity cards” of ginseng radix et rhizoma; Table S3: “Matrix identity cards” of panacis quinquefolii radix.

## Abbreviations

The following abbreviations are used in this manuscript:

CC	contrast credibility
DM	data matrix
GR	ginseng radix et rhizoma
GRS	GR blind samples
LC	liquid chromatography
MIC	matrix identity cards
MS	mass spectrometry
PAS	positive adulterated sample
PR	panacis quinquefolii radix
TCMs	traditional Chinese medicines
UPLC-QTOF-MS	ultra-performance liquid chromatography–quadrupole time-of-flight tandem mass spectrometry

## References

[1] Mo P., Gao B., Wang R., Huang S., Chen Q., Li M., Wu J., Zhang S., Chen J. (2025). Knowledge Mapping and Global Research Trends of Ginseng Polysaccharides: A Bibliometric Analysis with Visualizations from 1985 to 2023. Drug Des. Devel. Ther..

[2] Chen W., Yao P., Vong C.T., Li X., Chen Z., Xiao J., Wang S., Wang Y. (2021). Ginseng: A bibliometric analysis of 40-year journey of global clinical trials. J. Adv. Res..

[3] Liu H., Lu X., Hu Y., Fan X. (2020). Chemical constituents of Panax ginseng and Panax notoginseng explain why they differ in therapeutic efficacy. Pharmacol. Res..

[4] Fan W., Fan L., Wang Z., Mei Y., Liu L., Li L., Yang L., Wang Z. (2024). Rare ginsenosides: A unique perspective of ginseng research. J. Adv. Res..

[5] Kim Y., Lee J.W., Jo I.H., Kwon N., Kim D., Chung J.W., Bang K.H., Sung J. (2022). Volatile Compositions of Panax ginseng and Panax quinquifolium Grown for Different Cultivation Years. Foods.

[6] Ran X., Dou D., Chen H., Ren G. (2022). The correlations of adverse effect and tonifying effect of ginseng medicines. J. Ethnopharmacol..

[7] Jurica K., Brčić Karačonji I., Lasić D., Bursać Kovačević D., Putnik P. (2021). Unauthorized Food Manipulation as a Criminal Offense: Food Authenticity, Legal Frameworks, Analytical Tools and Cases. Foods.

[8] Wang H.P., Zhang Y.B., Yang X.W., Zhao D.Q., Wang Y.P. (2016). Rapid characterization of ginsenosides in the roots and rhizomes of Panax ginseng by UPLC-DAD-QTOF-MS/MS and simultaneous determination of 19 ginsenosides by HPLC-ESI-MS. J. Ginseng Res..

[9] Yang Y., Ju Z., Yang Y., Zhang Y., Yang L., Wang Z. (2021). Phytochemical analysis of Panax species: A review. J. Ginseng Res..

[10] Yoon D., Shin W.C., Oh S.M., Choi B.R., Young Lee D. (2022). Integration of multiplatform metabolomics and multivariate analysis for geographical origin discrimination of Panax ginseng. Food Res. Int..

[11] Xu X.F., Cheng X.L., Lin Q.H., Li S.S., Jia Z., Han T., Lin R.C., Wang D., Wei F., Li X.R. (2016). Identification of mountain-cultivated ginseng and cultivated ginseng using UPLC/oa-TOF-MSE with a multivariate statistical sample-profiling strategy. J. Ginseng Res..

[12] Deng J., Liu T., Xia C., Tong L., Gu C., Shi Z., Yang Y., Zhan R., Xiang Z., Chen J. (2025). Characteristics of Polyphenols of Black Hulless Barley Bran and Its Anti-Diabetic Activity. Foods.

[13] Bi S., Li N., Gong G., Gao P., Zhu J., Abulikemu B. (2025). Elucidating Volatile Flavor Profiles and Metabolic Pathways in Northern Pike (Esox lucius) During Superchilled Storage: A Combined UPLC-Q-TOF/MS and GC-MS Approach. Foods.

[14] Wang X., Li M., Zhang Y., Zhang J., Jing W., Guo X., Cheng X., Wei F. (2025). Identification of Rubiae Radix et Rhizoma and Its Adulterants Based on “Mass Spectrometry Matrix”. Anal. Sci. Adv..

[15] Zhang J., He F., Wang X., Jing W., Li M., Guo X., Cheng X., An F., Wei F. (2024). Identification of Ginseng Radix et Rhizoma, Panacis Quinquefolii Radix, Notoginseng Radix et Rhizoma, and Platycodonis Radix Based on UHPLC-QTOF-MS and “Matrix Characteristics”. Separations.

[16] Lievens A., Paracchini V., Pietretti D., Garlant L., Maquet A., Ulberth F. (2021). DNA Accounting: Tallying Genomes to Detect Adulterated Saffron. Foods.

[17] Liu Y., Wang X., Wang L., Chen X., Pang X., Han J. (2016). A Nucleotide Signature for the Identification of American Ginseng and Its Products. Front. Plant Sci..

[18] Kesanakurti P., Ragupathy S., Faller A.C., Shanmughanandhan D., Buongiorno F., Della Noce I., Lu Z., Zhang Y., Newmaster S.G. (2021). Development of Hydrolysis Probe-Based qPCR Assays for Panax ginseng and Panax quinquefolius for Detection of Adulteration in Ginseng Herbal Products. Foods.

[19] National Pharmacopoeia Commission (2020). Pharmacopoeia of the People’s Republic of China: A part I.

[20] Liao J., Zhang Y., Zhang W., Zeng Y., Zhao J., Zhang J., Yao T., Li H., Shen X., Wu G. (2023). Different software processing affects the peak picking and metabolic pathway recognition of metabolomics data. J. Chromatogr. A..

[21] Zhang L., Han T., Wang X., Zhang Y., Zhang J., Jing W., Li M., Cheng X., Wei F. (2025). Identification and Adulteration Evaluation of Rubiae Radix Et Rhizoma and Its Common Adulterants Based on LC-MS and Chemometrics. Molecules.

[22] Wang X., He F., Wu H., Jing W., Li M., Guo X., Cheng X., Wei F. (2025). Analysis of Saposhnikoviae Radix and Libonotidis Radix Based on “Characteristic Ion Set” and Chemometrics. J. Mass. Spectrom..

[23] Wang X., Zhang J., He F., Jing W., Guo X., Li M., Yu K., Yang J., Cheng X., Wei F. (2025). Traceability and Adulteration Analysis of Citri Reticulatae Pericarpium Based on “Digital Identity Card” and UHPLC-QTOF-MS Analysis. Food Sci. Nutr..

[24] Zhang L., Wang P., Li S., Wu D., Zhong Y., Li W., Xu H., Huang L. (2023). Differentiation of Mountain- and Garden-Cultivated Ginseng with Different Growth Years Using HS-SPME-GC-MS Coupled with Chemometrics. Molecules.

[25] Zhang W., Zhang C., Cao L., Liang F., Xie W., Tao L., Chen C., Yang M., Zhong L. (2023). Application of digital-intelligence technology in the processing of Chinese materia medica. Front. Pharmacol..

[26] Zhou W., Liu D., Yi Z., Lei Y., Zhang Z., Deng Y., Tan Y. (2024). Web-Based Platform for Systematic Reviews and Meta-Analyses of Traditional Chinese Medicine: Platform Development Study. JMIR Form. Res..

[27] Zhu J., Liu X., Gao P. (2025). Digital intelligence technology: New quality productivity for precision traditional Chinese medicine. Front. Pharmacol..

[28] Yao B.L., Wang R.R., Zhang M., Wei J.J., Yin Y.Z., Han H.Q. (2023). Simultaneous Determination of Panax quinquefolium Illegally Added in Renshen Jianpi Pills and Renshen Guipi Pills by UPLC-MS/MS. Mod. Chin. Med..

[29] Ahmed S., Kennedy G., Crum J., Vogliano C., McClung S., Anderson C. (2021). Suitability of Data-Collection Methods, Tools, and Metrics for Evaluating Market Food Environments in Low- and Middle-Income Countries. Foods.

