# Supplementary material for: A New Method for Identification of Ginseng Radix et Rhizoma Adulterated with Panacis Quinquefolii Radix
Source: Foods. 2025 Oct 20;14(20):3566. doi: 10.3390/foods14203566 (PMC12564425; doi:10.3390/foods14203566)
Supplement: Supplementary file 1 [file foods-14-03566-s001.zip › foods-3891739-supplementary.pdf]

# A new method for identification of Ginseng radix et rhizoma adulterated with Panacis quinquefolii radix

Table S1. The detailed information of herbal materials

| Herbal materials | Batches | Sample collection unit | Use | Place of origin |
|---|---|---|---|---|
| Ginseng radix et rhizoma | GR01 | National Institutes for Food and Drug Control | Identification verification; mixed sample | Shanxi, China |
| Ginseng radix et rhizoma | GR02 | National Institutes for Food and Drug Control | Identification verification; mixed sample | Liaoning, China |
| Ginseng radix et rhizoma | GR03 | National Institutes for Food and Drug Control | "Matrix identity cards"; mixed sample | Jinlin, China |
| Ginseng radix et rhizoma | GR04 | National Institutes for Food and Drug Control | "Matrix identity cards"; mixed sample | Heilongjiang, China |
| Ginseng radix et rhizoma | GR05 | National Institutes for Food and Drug Control | Identification verification; mixed sample | Shandong, China |
| Ginseng radix et rhizoma | GR06 | National Institutes for Food and Drug Control | "Matrix identity cards"; mixed sample | Hebei, China |
| Ginseng radix et rhizoma | GR07 | National Institutes for Food and Drug Control | "Matrix identity cards"; mixed sample | Liaoning, China |
| Ginseng radix et rhizoma | GR08 | National Institutes for Food and Drug Control | Identification verification; mixed sample | Gansu, China |
| Ginseng radix et rhizoma | GR09 | National Institutes for Food and Drug Control | Identification verification; mixed sample | Sichuan, China |
| Ginseng radix et rhizoma | GR10 | National Institutes for Food and Drug Control | Identification verification; mixed sample | Yunnan, China |
| Ginseng radix et rhizoma | GR11 | National Institutes for Food and Drug Control | Identification verification; mixed sample | Jinlin, China |
| Ginseng radix et rhizoma | GR12 | National Institutes for Food and Drug Control | "Matrix identity cards"; mixed sample | Shandong, China |
| Ginseng radix et rhizoma | GR13 | National Institutes for Food and Drug Control | Identification verification; mixed sample | Anhui, China |
| Ginseng radix et rhizoma | GR14 | National Institutes for Food and Drug Control | Identification verification; mixed sample | Liaoning, China |
| Ginseng radix et rhizoma | GR15 | National Institutes for Food and Drug Control | Identification verification; mixed sample | Jiangxi, China |
| Panacis quinquefolii radix | PR01 | National Institutes for Food and Drug Control | "Matrix identity cards"; mixed sample | Shandong, China |
| Panacis quinquefolii radix | PR02 | National Institutes for Food and Drug Control | Identification verification; mixed sample | Jilin, China |
| Panacis quinquefolii radix | PR03 | National Institutes for Food and Drug Control | "Matrix identity cards"; mixed sample | Fujian, China |
| Panacis quinquefolii radix | PR04 | National Institutes for Food and Drug Control | "Matrix identity cards"; mixed sample | Beijing, China |
| Panacis quinquefolii radix | PR05 | National Institutes for Food and Drug Control | Identification verification; mixed sample | Anhui,China |
| Panacis quinquefolii radix | PR06 | National Institutes for Food and Drug Control | Identification verification; mixed sample | Yunnan, China |

| | | | | |
|---|---|---|---|---|
| Panacis quinquefolii radix | PR07 | National Institutes for Food and Drug Control | Identification verification; mixed sample | Shandong, China |
| Panacis quinquefolii radix | PR08 | National Institutes for Food and Drug Control | "Matrix identity cards"; mixed sample | Yunnan, China |
| Panacis quinquefolii radix | PR09 | National Institutes for Food and Drug Control | "Matrix identity cards"; mixed sample | Shan'Xi, China |
| Panacis quinquefolii radix | PR10 | National Institutes for Food and Drug Control | Identification verification; mixed sample | Hebei, China |
| Panacis quinquefolii radix | PR11 | National Institutes for Food and Drug Control | Identification verification; mixed sample | Liaoning, China |
| Panacis quinquefolii radix | PR12 | National Institutes for Food and Drug Control | Identification verification; mixed sample | Beijing, China |
| Panacis quinquefolii radix | PR13 | National Institutes for Food and Drug Control | Identification verification; mixed sample | Jilin, China |
| Panacis quinquefolii radix | PR14 | National Institutes for Food and Drug Control | Identification verification; mixed sample | Fujian, China |
| Panacis quinquefolii radix | PR15 | National Institutes for Food and Drug Control | Identification verification; mixed sample | Anhui,China |
| 0 % PR | PAS01 | self-preparation | Identification verification | — |
| 5 % PR | PAS02 | self-preparation | Identification verification | — |
| 5 % PR | PAS03 | self-preparation | Identification verification | — |
| 5 % PR | PAS04 | self-preparation | Identification verification | — |
| 5 % PR | PAS05 | self-preparation | Identification verification | — |
| 5 % PR | PAS06 | self-preparation | Identification verification | — |
| 5 % PR | PAS07 | self-preparation | Identification verification | — |
| 10 % PR | PAS08 | self-preparation | Identification verification | — |
| 20 % PR | PAS09 | self-preparation | Identification verification | — |
| 50 % PR | PAS10 | self-preparation | Identification verification | — |
| 100 % PR | PAS11 | self-preparation | Identification verification | — |
| GR blind samples | GRS01 | Herbal Markets | Identification analysis | Beijing, China |
| GR blind samples | GRS02 | Herbal Markets | Identification analysis | Beijing, China |
| GR blind samples | GRS03 | Herbal Markets | Identification analysis | Beijing, China |
| GR blind samples | GRS04 | Herbal Markets | Identification analysis | Jilin, China |
| GR blind samples | GRS05 | Herbal Markets | Identification analysis | Jilin, China |

| | | | | |
|---|---|---|---|---|
| GR blind samples | GRS06 | Herbal Markets | Identification analysis | Jilin, China |
| GR blind samples | GRS07 | Herbal Markets | Identification analysis | Liaoning, China |
| GR blind samples | GRS08 | Herbal Markets | Identification analysis | Shanxi, China |
| GR blind samples | GRS09 | Herbal Markets | Identification analysis | Hebei, China |
| GR blind samples | GRS10 | Herbal Markets | Identification analysis | Gansu, China |

Table S2. The "matrix identity cards" of ginseng radix et rhizoma

| $t_R$ | *m/z* | I | $t_R$ | *m/z* | I |
|---|---|---|---|---|---|
| 18.56 | 1193.60 | 494893 | 15.52 | 1439.65 | 23562 |
| 18.14 | 1165.60 | 441930 | 5.52 | 913.45 | 23271 |
| 19.16 | 1165.60 | 433791 | 17.92 | 1405.59 | 23086 |
| 17.92 | 1255.64 | 373822 | 17.12 | 1344.62 | 23067 |
| 17.12 | 1295.64 | 311946 | 20.68 | 1212.58 | 22709 |
| 18.44 | 1393.61 | 233037 | 11.22 | 675.11 | 22474 |
| 19.19 | 1212.58 | 214943 | 17.33 | 1423.62 | 22320 |
| 17.91 | 1307.61 | 212549 | 16.53 | 1325.64 | 22141 |
| 16.58 | 1307.61 | 211914 | 16.77 | 1219.63 | 22135 |
| 21.78 | 1031.55 | 152107 | 17.89 | 1250.61 | 21644 |
| 19.90 | 1295.64 | 130743 | 18.44 | 1394.11 | 20948 |
| 8.94 | 1467.82 | 125796 | 13.46 | 1297.71 | 20830 |
| 17.12 | 1393.61 | 115753 | 13.46 | 845.78 | 20705 |
| 18.45 | 1344.62 | 94231 | 11.67 | 983.46 | 20483 |
| 17.92 | 650.32 | 91135 | 10.45 | 1328.74 | 20108 |
| 20.18 | 1295.63 | 86265 | 11.89 | 958.52 | 19908 |
| 18.85 | 1194.61 | 63831 | 15.48 | 821.41 | 19856 |
| 14.22 | 983.46 | 63608 | 16.58 | 1256.14 | 19775 |
| 14.22 | 887.50 | 61067 | 17.92 | 1232.64 | 19643 |
| 16.76 | 1196.63 | 58627 | 20.73 | 1249.59 | 19616 |
| 15.61 | 1423.62 | 57371 | 21.60 | 1118.05 | 19592 |
| 12.64 | 1069.46 | 56657 | 17.91 | 1259.12 | 19591 |
| 18.44 | 1296.14 | 52453 | 16.33 | 1337.62 | 18962 |
| 8.68 | 1248.72 | 48311 | 20.40 | 1249.59 | 18636 |
| 16.78 | 1337.62 | 47477 | 18.64 | 1255.64 | 18628 |
| 14.22 | 885.49 | 45176 | 19.52 | 1307.61 | 18614 |
| 19.90 | 1393.61 | 44649 | 16.58 | 1405.59 | 18248 |
| 11.88 | 886.49 | 42813 | 16.57 | 1245.61 | 17743 |
| 18.44 | 1287.62 | 40741 | 22.09 | 1215.52 | 17709 |
| 13.46 | 1248.72 | 37481 | 18.60 | 1325.65 | 17588 |
| 18.44 | 1299.66 | 36857 | 8.70 | 1398.47 | 17332 |
| 19.17 | 1262.07 | 36819 | 11.84 | 1080.53 | 17246 |
| 11.17 | 577.14 | 36450 | 21.13 | 1297.65 | 17219 |
| 17.32 | 1308.61 | 34880 | 17.89 | 1224.62 | 17135 |
| 21.60 | 1166.53 | 34565 | 17.91 | 1245.61 | 16985 |
| 18.44 | 1336.61 | 33807 | 16.57 | 1232.64 | 16421 |
| 18.32 | 1307.61 | 33615 | 16.77 | 1271.60 | 16156 |
| 13.46 | 1245.74 | 32415 | 8.93 | 1326.38 | 16056 |
| 20.18 | 1393.61 | 31165 | 20.69 | 1359.54 | 16055 |
| 15.52 | 1387.68 | 29223 | 18.44 | 1491.59 | 15938 |
| 10.42 | 1081.44 | 28186 | 18.56 | 1113.57 | 15868 |
| 19.51 | 1255.64 | 27784 | 11.19 | 983.46 | 15637 |
| 15.96 | 1427.68 | 26580 | 13.46 | 1271.73 | 15150 |
| 19.13 | 1359.55 | 26567 | 18.13 | 1310.56 | 15040 |
| 16.58 | 1259.12 | 25388 | 18.44 | 1385.60 | 15029 |
| 11.19 | 885.49 | 25127 | 16.94 | 1307.61 | 14826 |
| 13.47 | 835.46 | 25083 | 19.56 | 1262.07 | 14792 |
| 18.51 | 1209.62 | 24692 | 17.91 | 1308.11 | 14726 |
| 17.92 | 1256.14 | 24637 | 18.64 | 1307.61 | 14707 |
| 16.94 | 1255.64 | 23956 | 18.16 | 1164.94 | 14553 |

Table S3. The "matrix identity cards" of panacis quinquefolii radix

| $t_R$ | *m/z* | I | $t_R$ | *m/z* | I |
|---|---|---|---|---|---|
| 13.71 | 845.50 | 672013 | 20.96 | 989.53 | 10227 |
| 21.70 | 1464.83 | 319208 | 14.30 | 1187.56 | 10215 |
| 21.71 | 968.55 | 122430 | 4.92 | 993.53 | 10001 |
| 21.71 | 992.05 | 107196 | 18.99 | 1497.82 | 9900 |
| 21.70 | 1441.83 | 71206 | 0.44 | 345.08 | 9600 |
| 17.40 | 1087.54 | 69526 | 13.71 | 862.48 | 9077 |
| 8.97 | 1468.32 | 66380 | 23.26 | 1221.75 | 9035 |
| 19.02 | 839.44 | 49769 | 18.63 | 1261.58 | 8990 |
| 18.29 | 971.49 | 49600 | 12.00 | 600.29 | 8973 |
| 21.71 | 991.87 | 48199 | 8.65 | 1416.80 | 8799 |
| 21.05 | 1165.60 | 36756 | 23.16 | 1249.66 | 8647 |
| 21.69 | 1468.32 | 31140 | 17.67 | 1291.57 | 8528 |
| 18.27 | 925.98 | 29367 | 14.77 | 1007.54 | 8526 |
| 13.70 | 845.78 | 27367 | 22.88 | 674.35 | 8484 |
| 19.00 | 1332.74 | 25156 | 16.88 | 1457.11 | 8336 |
| 21.69 | 1017.53 | 24915 | 13.11 | 831.47 | 8282 |
| 20.55 | 1418.83 | 24171 | 17.40 | 566.27 | 8197 |
| 14.55 | 1249.59 | 23418 | 24.53 | 1277.69 | 8128 |
| 13.69 | 835.46 | 23319 | 21.07 | 1099.53 | 8063 |
| 8.62 | 1187.61 | 23148 | 14.72 | 887.50 | 8014 |
| 16.16 | 1187.56 | 22658 | 18.68 | 1163.59 | 7903 |
| 18.29 | 1388.73 | 22318 | 20.55 | 996.03 | 7886 |
| 12.24 | 1025.55 | 22243 | 21.02 | 1163.59 | 7793 |
| 15.29 | 1087.53 | 20908 | 20.04 | 620.56 | 7565 |
| 17.62 | 1158.59 | 20013 | 21.69 | 1473.33 | 7558 |
| 16.89 | 1325.03 | 19562 | 23.26 | 830.01 | 7494 |
| 18.35 | 620.31 | 19497 | 13.90 | 863.50 | 7387 |
| 19.26 | 1235.61 | 18233 | 21.70 | 1465.71 | 7339 |
| 13.48 | 699.43 | 18058 | 17.35 | 1227.59 | 7284 |
| 13.19 | 606.29 | 18011 | 20.50 | 1485.81 | 7211 |
| 13.71 | 1248.72 | 17620 | 18.67 | 1164.59 | 6921 |
| 21.69 | 1459.82 | 17077 | 20.65 | 989.53 | 6910 |
| 19.01 | 1190.66 | 16709 | 19.26 | 1233.63 | 6878 |
| 8.64 | 1016.55 | 15888 | 15.54 | 1005.53 | 6823 |
| 17.43 | 1021.51 | 14896 | 24.17 | 1254.69 | 6765 |
| 17.00 | 1088.04 | 14795 | 9.58 | 847.50 | 6606 |
| 13.70 | 846.02 | 14505 | 23.27 | 829.79 | 6583 |
| 13.13 | 1025.55 | 13672 | 19.24 | 1279.63 | 6441 |
| 20.53 | 1459.82 | 13028 | 20.05 | 1135.59 | 6430 |
| 21.70 | 986.53 | 12890 | 20.52 | 1070.58 | 6428 |
| 22.88 | 1303.71 | 12502 | 20.53 | 963.53 | 6302 |
| 12.00 | 1155.58 | 12288 | 18.35 | 1247.58 | 6272 |
| 16.13 | 1087.53 | 12103 | 18.98 | 935.51 | 6219 |
| 20.50 | 1093.58 | 11888 | 13.36 | 821.45 | 6174 |
| 17.39 | 1074.55 | 11731 | 16.73 | 887.50 | 6032 |
| 21.69 | 1008.54 | 11698 | 5.04 | 717.44 | 5906 |
| 12.96 | 887.50 | 11142 | 14.72 | 885.48 | 5882 |
| 15.34 | 1007.54 | 10635 | 18.59 | 1136.10 | 5856 |
| 17.40 | 1185.51 | 10496 | 16.88 | 1391.07 | 5848 |
| 16.02 | 1306.69 | 10416 | 14.27 | 1005.53 | 5743 |
